# Between Dysbiosis, Maternal Immune Activation and Autism: Is There a Common Pathway?

**DOI:** 10.3390/nu16040549

**Published:** 2024-02-16

**Authors:** Maria Suprunowicz, Natalia Tomaszek, Agata Urbaniak, Klaudia Zackiewicz, Stefan Modzelewski, Napoleon Waszkiewicz

**Affiliations:** Department of Psychiatry, Medical University of Bialystok, pl. Wołodyjowskiego 2, 15-272 Białystok, Poland; 37923@student.umb.edu.pl (M.S.); 38778@student.umb.edu.pl (N.T.); 38780@student.umb.edu.pl (A.U.); 38791@student.umb.edu.pl (K.Z.); napoleon.waszkiewicz@umb.edu.pl (N.W.)

**Keywords:** autism spectrum disorders, gut microbiota, brain–gut axis, maternal immune activation (MIA), gastrointestinal, delivery, dysbiosis, neurodevelopment, microglia, short-chain fatty acids (SCFA)

## Abstract

Autism spectrum disorder (ASD) is a neuropsychiatric condition characterized by impaired social interactions and repetitive stereotyped behaviors. Growing evidence highlights an important role of the gut–brain–microbiome axis in the pathogenesis of ASD. Research indicates an abnormal composition of the gut microbiome and the potential involvement of bacterial molecules in neuroinflammation and brain development disruptions. Concurrently, attention is directed towards the role of short-chain fatty acids (SCFAs) and impaired intestinal tightness. This comprehensive review emphasizes the potential impact of maternal gut microbiota changes on the development of autism in children, especially considering maternal immune activation (MIA). The following paper evaluates the impact of the birth route on the colonization of the child with bacteria in the first weeks of life. Furthermore, it explores the role of pro-inflammatory cytokines, such as IL-6 and IL-17a and mother’s obesity as potentially environmental factors of ASD. The purpose of this review is to advance our understanding of ASD pathogenesis, while also searching for the positive implications of the latest therapies, such as probiotics, prebiotics or fecal microbiota transplantation, targeting the gut microbiota and reducing inflammation. This review aims to provide valuable insights that could instruct future studies and treatments for individuals affected by ASD.

## 1. Introduction

Autism spectrum disorder (ASD) is a neurodevelopmental condition defined by early deficits in social interaction/communication and repetitive stereotyped behaviors [1]. The multifactorial etiology of ASD includes both genetics and environmental factors. Genetic mutations, maternal immune activation, and environmental triggers such as toxicants, insecticides, infections, and medications are involved [2]. ASD consists of frequent gastrointestinal (GI) symptoms with variable prevalence, including chronic diarrhea, constipation, abdominal bloating, and discomfort [3].

Correlations between GI dysfunction and worsened behavioral symptoms have become evidence of brain–gut axis pathophysiology in ASD patients and suggest the intestinal microbiome as a significant factor. Researchers observed changes in the ASD gut microbiome compared to typically developed children; however, they can result from differences in diet, medical comorbidities, and geographic location [4]. Thus, further work is needed to better understand the concept of the microbiota–gut–brain axis. The gut microbiome is shaped from the earliest years of life and regulates important processes such as digestion and immune response [5].

The number of intestinal bacteria exceeds the number of human cells and genes [6]. Therefore, there is no doubt that its role is crucial in the proper functioning of the human body. Changes in the mother’s microbiome influence offspring gut microbial structure and composition [7]. Many data confirm the interaction of microbiota in pregnancy and the prenatal and newborn period [8].

Infections or injuries during pregnancy can induce inflammation, subsequently impacting fetal brain development [9]. Maternal immune activation (MIA) is considered to be a disease primer, making offspring more susceptible to other risk factors, like genetic and environmental ones [10]. Pregnant women exposed to MIA have been shown to have pathological activation of specific interleukins, which promotes abnormal cortical development and ASD-like phenotypes in the offspring [11].

In this review, we will compile the results of the numerous articles and studies to examine the role of the gut microbiome in developing ASD via the gut–brain axis. We will focus especially on the mother’s microflora and its influence on prenatal brain development.

## 2. Methods

The PubMed and Web of Science databases were searched using the following key words: “autism spectrum disorder”, “ASD”, “gut microbiota”, “dysbiosis”, “brain–gut axis”, “leaky gut”, “SCFA”, “maternal immune activation”, “MIA”, “neuroinflammation”, “microglia”, “IL-6”, “IL-17a”, “obesity”, “high-fat diet”, “maternal factors”, “therapy”, “delivery”, “gastrointestinal”, “prebiotics”, “probiotics”, and “fecal microbiota transplantation”, as well as combinations of these terms. We included relevant articles to assess the potential correlation of changes in maternal microflora with a child’s risk of developing autism.

## 3. The Mode of Delivery and Microbiota Transfer

Proper human development is an intricate process involving numerous genetic and environmental factors [12]. The gut microbiome has emerged as one of the crucial components due to its undoubted influence on health throughout the entire life [13].

The impaired balance of the gut flora in infancy is linked to an increased risk of numerous diseases, especially of immunological origins, like asthma [14] and allergies [15]. Moreover, disruptions in this balance have been associated with a range of mental and neurological disorders, such as depression [16,17], anxiety [18], schizophrenia [19,20], Parkinson’s disease [21], Alzheimer’s disease [22], and autism [23].

According to the well-established doctrine, microbiota acquisition begins at birth, as a result of exposition to the maternal birth canal environment [23]. However, this statement has recently been reassessed by a limited number of studies confirming the presence of microorganisms in the placenta [24,25,26]. These results are still the subject of debate in the scientific community, and there is no clear conclusion [27].

Kennedy et al. conducted a multidisciplinary evaluation of similar studies supporting the evidence of microbial presence in prenatal intrauterine locations. Based on their findings, it is more likely that the observed microbial signals were the effect of contamination during the collection and processing of samples and data, rather than genuine microbial colonization. Analyzed studies frequently indicate the presence of microorganisms, widely known as common contaminants such as *Bradyrhizobium* and *Micrococcus.* The researchers emphasize the challenge of distinguishing relevant microbial signals from contaminating noise in low-biomass samples, which can lead to misconceptions about tissue sterility. Therefore, they highlight the importance of following a trans-disciplinary approach, considering biological, ecological, and mechanistic explanations, when studying low-biomass samples. This approach should facilitate the proper interpretation of findings and address the challenges posed by contamination [28]. 

A characteristic microbiome has been identified in the placenta, the amniotic fluid, and the fetus in healthy pregnancies [24]. Nonetheless, it is unclear when the first fetal exposition to bacteria is and where they come from [8]. Modification in placental microbiota may be related to infections, including urinary tract infections resulting in placental enrichment of *Streptococcus*, *Arthrobacter*, *Klebsiella*, and *Acinetobacter* [24]. 

### 3.1. The Mode of Delivery and Microbiota Transmission

After birth, the diversity of microbiota changes due to the contribution of multiple factors such as skin-to-skin contact [29], breastfeeding [30], diet [31], antibiotic administration [32], and other environmental exposures [33,34,35]. Nevertheless, the mode of delivery is considered one of the most significant determinants influencing the heterogeneity of gut microorganisms in early life [36].

The majority of the studies show numerous differences between vaginal (VD) and cesarean section (CS) babies in terms of composition, amount, and maturation onset of gut microbiota [23,36,37,38,39,40,41]. CS children are more likely to be inhabited by bacterial species similar to the mother’s skin surface (e.g., *Staphylococcus*, *Corynebacterium*, and *Propionibacterium* spp.) [23]. Their microbiota is more abundant in potentially pathogenic species like *Enterococcus*, *Enterobacter*, and *Klebsiella*, usually associated with hospital units [37,38].

On the other hand, VD children inherit microbiota closely resembling the mother’s vaginal environment [23]. Such neonates have more prevalent and diverse communities of Lactobacillus and Bifidobacterium taxa [36,39], known for their positive impact on infant’s health (29). Moreover, the microbiota composition (at the genus and phylum levels) remains stable during VD children’s development as opposed to CS [40]. Over time, those differences diminish and become less noticeable in 6–8 weeks after birth [39,42]. This brief period is crucial for proper neurodevelopment. It overlaps with the initiation of the most significant elongation of axons and dendrite branching, alongside the beginning of accelerated synaptogenesis [43]. 

### 3.2. Changes in Gut Microflora in Autism

Gut dysbiosis is a health complication with greater prevalence in ASD patients compared to neurotypical individuals [44]. ASD-diagnosed individuals have less diverse gut microbiota, with the main components consisting of *Bacteroidetes*, *Parabacteroides*, *Faecalibacterium*, *Phascolarctobacterium*, *Lactobacillus*, *Clostridioides*, *Desulfovibrio*, *Caloramator*, and *Sarcina* compared to the control group [45,46,47]. Additionally, decreased levels of *Coprococcus* and *Bifidobacterium* were discovered [46]. Another data analysis revealed a reduction in *Prevotella*, *Coprococcus*, *Enterococcus*, *Lactobacillus*, *Streptococcus*, *Lactococcus*, *Staphylococcus*, *Ruminococcus*, and *Bifidobacterium* species and higher levels of *Clostridia* and *Desulfovibrio* [48]. Nonetheless, not all studies confirm this relationship, i.e., research on ASD patients and their neurotypical siblings indicated no significant differences in gut microbiota diversity [49]. 

However, microbiota disturbances are still frequently linked to ASD. For example, intensive antibiotic therapy, repeatedly used in ASD-diagnosed children might result in the overgrowth of *Desulfovibrio* bacteria [50]. The involvement of *Desulfovibrio* in ASD pathogenesis is underscored through its production of Lipopolysaccharide (LPS) and its known role in promoting inflammation [50]. Tomova et al. in a study involving a small group of ASD-diagnosed children demonstrated a significant association between autism severity and the abundance of *Desulfovibrio* spp. [51].

Moreover, ASD patients typically exhibit decreased levels of *Lactobacillus* spp. [52]. It is worth noticing that attempts at recolonization with *Lactobacillus reuteri* have shown partial alleviation of intestine inflammation caused by LPS. Additionally, supplementation with *Bacteroides fragilis* has been found to reduce gut permeability [53]. 

The gut microbiota not only encompasses bacteria but also includes fungi. A good example is *Candida* spp., which has been proclaimed to take part in ASD pathogenesis [54]. Elevated concentrations of *Candida* yeasts have been observed in fecal samples from individuals with ASD [55]. Maintaining an appropriate concentration of *Lactobacillus* spp. prevents the overgrowth of *Candida*; however, autistic individuals exhibit reduced numbers of *Lactobacillus* spp. [56]. Additionally, an excessive *Candida* population impedes re-establishment with commensal microorganisms [57]. The proliferation of *Candida* yeasts results in an increased production of ammonia and toxins, which studies have linked to the exacerbation of autistic behaviors [58]. Furthermore, Candida overgrowth may lead to the malabsorption of minerals and carbohydrates [57]. Therefore, addressing the balance of gut microbiota, particularly managing *Candida levels* and promoting the presence of beneficial bacteria, becomes essential research interest in the context of ASD.

Research on microbiome changes in autism is inconclusive. Differences may be influenced by individual variation in microflora composition, different ages and genders of subjects, severe eating restriction, food selectivity, disparities in the diet used or unknown factors.

## 4. Mode of Delivery and Autism Correlation

As the mode of delivery influences microbiota composition in early life, researchers focused on verifying its impact on the risk of autism.

Yip et al. analyzed records from the International Collaboration for Autism Registry Epidemiology (iCARE) database. Their study cohort consisted of 4,987,390 children born in 5 different countries (Norway, Sweden, Denmark, Finland, and Western Australia) and comprised 71,646 *C*-section deliveries. They ascertained that both—elective and emergency CS are associated with a higher risk of ASD in comparison to vaginal delivery [59]. Those findings were confirmed by more recent studies [60,61].

Furthermore, works by Chien et al., Huberman Samuel et al., and Yang et al. indicate that only CS performed under general anesthesia (GA) noticeably increases the risk of ASD. CS under regional anesthesia (RA) brought only an insignificantly higher risk than VD [62,63,64]. This might suggest that GA is a major factor contributing to the link between the mode of delivery and autism. However, those findings should be taken with caution due to several limitations of evaluated studies such as the omission of confounding factors, limited statistical power, and lack of sibling analysis. Moreover, the reason responsible for this phenomenon remains indistinct. Research based on human and animal models suggests that the administration of GA in early life might be the cause of neurotoxicity, which disturbs postpartum neurodevelopment [65]. These toxic effects might impact regions of synaptogenesis, which is especially accelerated in the first 6 months of life [43] and can be the cause of disruptions and delays in the subsequent development of other areas of the brain [66].

In addition, studies show that the general correlation between delivery mode and ASD might be related to confounding variables such as unknown genetic and environmental conditions. Curran et al. analyzed a large cohort of 2,697,315 children. Even though the general analysis proved that CS children are approximately 20% more likely to develop ASD after adjusting for sibling controls the association disappeared. Weaknesses of this study include the inability to verify the authenticity of the analyzed cases and determine whether the origin of confounding is a genetic or external factor. Furthermore, the sample size of the sibling control was significantly lower than the general study population [67].

In conclusion, most of the studies confirm that children delivered by cesarean section are more prone to the development of ASD. Additionally, the use of GA turned out to be one of the most feasible risk factors. Nevertheless, those findings must be taken cautiously as all confounders connected with CS should be considered. 

## 5. Microbiota Disruption and Its Potential Implications for ASD Development

Altered gut microbiota might impact brain development due to the existence of the gut–brain axis, which links the enteric and central nervous system (CNS) [68].

### 5.1. Bacteria as Producers of Short-Chain Fatty Acids (SCFAs)

**Figure 1:**
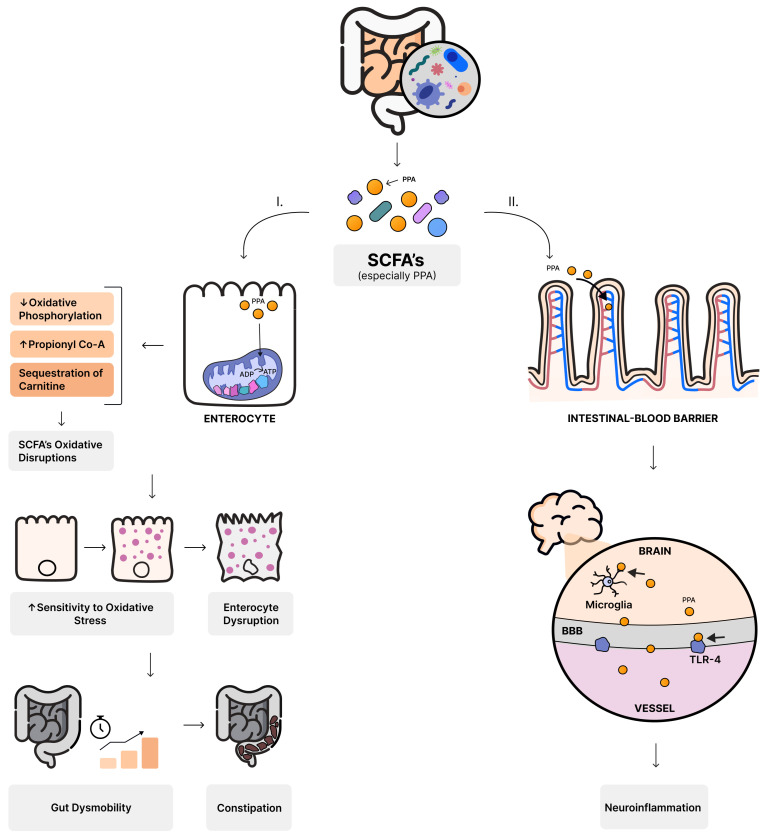
Diagram of the effect of overproduction of short-chain fatty acids (SCFAs). I—Increased production of one of the SCFAs, propionate (PPA), inhibits oxidative phosphorylation in the mitochondrion, increases propionyl-coenzyme A levels and causes carnitine sequestration. All of this can result in impaired SCFA oxidation, increasing sensitivity to oxidative stress and disrupting enterocyte function. The result can be gut dysmobility, manifested as constipation. II—Larger amounts of PPA can cross the intestinal–blood barrier and then the blood–brain barrier (BBB). Once across the barrier, they can be captured by microglia and alter its function, and bind to Toll like receptors TLR4 and activate the inflammatory response.

It has been discovered that a lack of proper microbiota results in immature and malfunctioning microglia [69]. One of the crucial reasons might be the fact that gut bacteria produce short-chain fatty acids (SCFAs), e.g., butyrate, propionate, acetate, and valerate, in the process of colonic fermentation [70]. Recognized as the primary signaling molecules between gut bacteria and the host, SCFAs exert their influence by binding to G-protein coupled receptors such as free fatty acid receptors (FFAR) [71]. SCFAs exhibit anti-inflammatory and anti-carcinogenic properties, regulate energy metabolism, hormonal secretion, and fortify the integrity of the gut barrier [72]. While normal levels of SCFAs regulate immune function in the gut, an overproduction can disrupt the gut balance and induce inflammation (Figure 1) [56]. 

SCFAs are associated with alterations in mitochondria function and epigenetic modulation of genes associated with ASD [73]. Moreover, they serve as substrates for energy production within mitochondria [74]. Elevated levels of propionic acid (PPA) may inhibit oxidative phosphorylation, increase levels of propionyl coenzyme A, and contribute to the sequestration of carnitine. These alterations can disrupt SCFA oxidation and heighten sensitivity to oxidative stress [75]. ASD-diagnosed individuals have been observed to exhibit mitochondrial dysfunction, potentially leading to disorganized enterocyte function and gut dysmotility [76]. These disturbances may manifest as constipation, a frequently reported issue among ASD patients [56]. 

One hypothesis of ASD pathogenesis underlines the crucial role of the spore-forming bacteria, particularly *Clostridioides* [77]. Toxins released by certain *Clostridioides* species can induce a proinflammatory response. These bacteria-origin toxins circulate in the bloodstream reaching the CNS and contributing to altered behaviors [78]. Antibiotic therapy has been suggested as a potential approach for enhancing the well-being of autistic patients [79].

In an environment abundant in sugar and carbohydrates coming from the diet, *Clostridioides* bacteria produce excessive amounts of SCFAs, especially propionic acid (PPA) [80]. PPA can cross the gut–blood barrier, but also the blood–brain barrier. Once in the CNS, PPA may be captured by glial cells, influencing physiological processes, health, and behavior [81]. Shultz et al. in a study performed on rats demonstrated that the intracerebroventricular injections of PPA resulted in behavioral changes similar to those observed in patients diagnosed with ASD [82]. Furthermore, SCFAs binding to Toll-like receptor 4 (TLR-4) influence the CNS and activate an inflammatory response [83]. 

Moreover, particularly one of the SCFAs—acetate—plays a critical role in microglia maturation [84]. Additionally, butyrate impacts the function of microglial cells—the immune cells of the CNS and participates in the regulation of neuroinflammation [85]. 

### 5.2. Microglia Dysregulation

**Figure 2:**
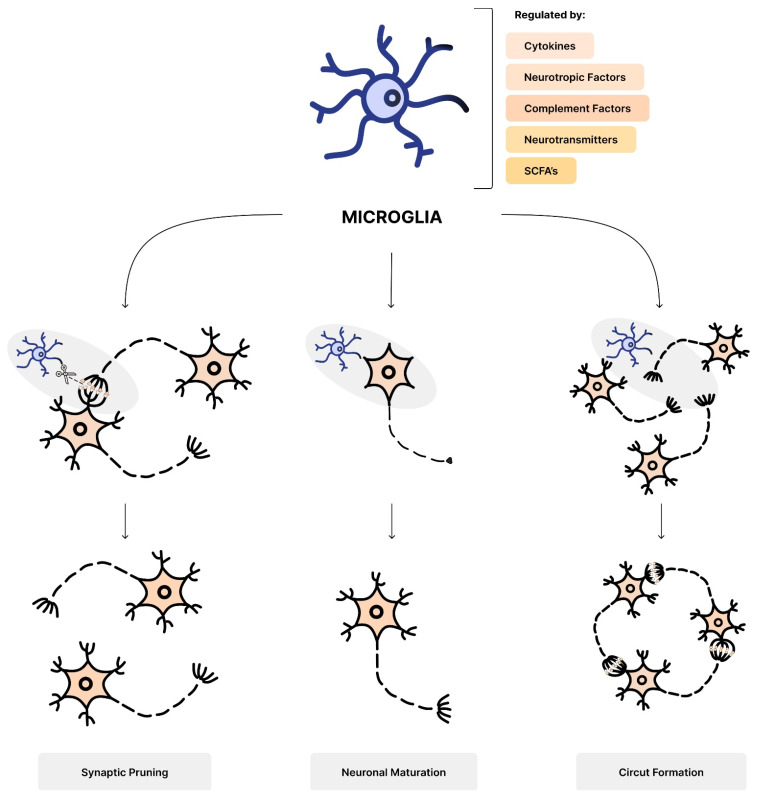
Diagram showing the role of microglia in brain formation. Microglia is regulated by cytokines, neurotropic factors, complement factors, neurotransmitters and short-chain fatty acids (SCFAs). Microglia: eliminate synaptic connections (synaptic pruning), influence morphological, electrophysiological and molecular characteristics of neurons (neuronal maturation) and they are responsible for neural circuit formation by synapse formation between neurons.

Microglia contribute to the maintenance of brain tissue homeostasis. They are regulated by various factors, such as cytokines, neurotrophic factors, complement factors, and neurotransmitters [86,87]. Microglia are responsible for synaptic pruning in the developing brain [88]. They also have a key role in neural circuit formation, neuronal differentiation, and maturation [89] (Figure 2). This is why its proper formation and activation are considered pivotal factors in neurogenesis.

Dysregulation of microglia activity is often suspected to be one of the potential mechanisms taking part in the development of ASD [90]. A post-mortem study by Vargas et al. observed that the brains of autistic patients could be characterized by increased microglial activation associated with neuroinflammation and expressed by elevated levels of cytokines such as Macrophage Chemoattractant Protein 1 (MCP-1) and Transforming growth factor beta 1 (TGF-1). Analysis showed that the microglial reaction was mainly spread out throughout cortical and subcortical regions and its presence was especially expressed in the cerebellum. Further, scientists observed areas with the formation of microglial nodules and clusters. According to the study, similar responses can be observed in neurodegenerative conditions such as Alzheimer’s disease (AD) and Parkinson’s disease (PD) [91].

Bifidobacterium and Lactobacillus are some of the major producers of SCFAs [92,93,94], which regulate microglia homeostasis [84,95]. Taking that into consideration, the decreased abundance of those microorganisms connected with CS [36,39] might be a cause of impaired microglia maturation and function, leading to a defective neuroimmunological response [84,95].

Zhan et al. discovered that insufficient microglial-mediated synaptic pruning, which relies on the targeted elimination of synapses, might be linked with autism-related behaviors [96]. Moreover, the study by Kim et al. confirms that mice with autophagy-deficient microglia present similar symptoms [97].

On the other hand, Seki et al. interlock Klebsiella overgrowth with disturbances of the gut–brain axis. According to the analysis, this phenomenon relates to the development of a specific composition of microorganisms promoting severe brain damage. They observed a negative correlation between increased levels of Klebsiella and neuroprotective factors such as BDNF and BDGF-BB [98]. Moreover, in the study by Lin et al. Klebsiella presented the ability to activate microglia, which released proinflammatory cytokines in response [99].

The link between microbiota, neurodevelopment, and ASD is still not well explored. Microglial development and synaptic forming stand out as the most promising mechanisms that might be linked with impaired delivery-acquired microbiota and the formation of autistic behaviors. Nevertheless, further research is required on this topic, focused on humans in addition to murine development.

## 6. The Gut–Brain Axis in ASD Patients

The gut–brain axis is a dynamic, bidirectional communication pathway connecting the central nervous system (CNS), consisting of the brain and the spinal cord, with the enteric nervous system (ENS), a complex network of neurons, interconnected small ganglia, submucosal and myenteric neuronal plexuses [100]. Often referred to as the “second brain”, the ENS can operate autonomously from the CNS to regulate gastrointestinal (GI) homeostasis [101]. 

Several underlying mechanisms, including neuronal, immune, and enteroendocrine pathways implement the link between the gastrointestinal tract and the brain [102]. Growing evidence indicates the existence of the gut–brain axis but also highlights the presence of the microbiota–gut–brain axis. Moreover, research has revealed that gut microbes can communicate with the brain [103]. Disturbances in the gut–brain axis play an important role in ASD pathogenesis and mainly include dysbiosis, increased permeability of the gut barrier, changes in neurotransmitters concentration, immune dysregulation, and neuroinflammation.

The major role is played by the vagus nerve, which comprises 80% of afferent fibers and 20% of efferent fibers [104]. Microbiota metabolites, gut hormones, and nutrients interact with the afferent branch of the vagus nerve and conduct signals to the CNS. Subsequently, the information in the CNS is evaluated and creates a response [105]. Efferent signals from the brain to the GI tract modulate the physiological functions of the guts and influence mobility, as well as the secretion of digestive enzymes [106]. 

Animal studies have shown the significance of the vagus nerve in mediating cerebro-intestinal communication. Researchers have demonstrated that administering *Lactobacillus rhamnosus* induced changes in γ-aminobutyric acid (GABA) receptor expression in mice and thereby decreased stress-induced corticosterone response, as well as reduced anxiety- and depressive-related behaviors [107]. Furthermore, this effect was absent in vagotomized individuals [107,108]. 

### 6.1. “Leaky Gut” in ASD

The proper functioning of the gut–brain axis relies fundamentally on the integrity of the barrier that separates the gut lumen from the tissues of the GI tract. The barrier comprises epithelial cells, a mucous layer, and tight junctions between cells [109]. These components prevent the organism from the entrance of harmful pathogens and toxins. A well-balanced microbiota composition fulfills an important role in maintaining the integrity of the GI barrier providing intercellular junctions [110]. For instance, *Lactobacillus* spp. takes part in maintaining tight junctions between cells and ASD patients are reported with decreased *Lactobacillus* spp. component in gut microbiota [55]. Moreover, in ASD brains studies show a disturbed expression of genes encoding proteins crucial for maintaining the integrity of the blood–brain barrier and gut barrier [111]. Increased permeability of the intestinal mucosa impairs the function of the gut barrier. That makes the immune system more sensitive to exogenous peptides from food or bacteria, toxins, and other metabolites [112,113]. This phenomenon is often referred to as “leaky gut” and researchers indicate its role in the complex pathogenesis of ASD [114]. Increased permeability results in elevated circulation of bacteria-derived lipopolysaccharide (LPS), triggering an inflammatory response marked by the expression of pro-inflammatory cytokines such as IL-1, IL-6, and IL-8 [91,115]. Furthermore, in ASD patients elevated levels of LPS are commonly reported [50]. Additionally, in a study performed on rats Kirsten et al. demonstrated that prenatal exposure to LPS resulted in autistic-like behaviors and hypoactivity of the dopaminergic system [116]. 

### 6.2. Enteroendocrine Cells and Neurotransmiters

Enteroendocrine cells (EECs) are crucial for the functioning of the gut–brain axis by acting as vital sensors within the GI tract and secreting various signaling molecules in response to different stimuli [117]. Situated in the intestinal epithelium, EECs intercommunicate with the vagus nerve afferent fibers by releasing serotonin (5-hydroxytryptamine, 5-HT) and activating 5-HT3 receptors [118]. Notably, the brain’s storage of tryptophan, a 5-HT precursor, is limited and more than 90% of 5-HT is stored and released by EECs [119]. Intestinal refilling of tryptophan is possible due to gut microorganisms such as *Bifidobacterium infantis* [120]. Serotonin has been identified in various microorganisms, including *Candida* spp., *Streptococcus* spp., *Escherichia* spp., and *Enterococcus* spp. [121].

The exact mechanism association between the disturbances in the serotoninergic system and occurring ASD is not fully understood. Disruption in the serotoninergic system during brain development may lead to long-term defects in overall brain function. Modifications to 5-HT neurons in the brainstem and synaptic and network alterations induce changes in projection areas linked to social behavior, such as the frontal cortex [122]. Marler et al. in a study performed on 82 children and adolescents with ASD observed increased whole blood serotonin concentration in 23% of participants [123]. One hypothesis suggests that an increased serotonin level during brain development might lead to further compensatory negative feedback, a reduced number of serotonin neurons, and decreased brain serotonin concentration [124,125,126]. Serotonin affects not only mood and brain function but also gastrointestinal secretion and mobility [127]. However, the research did not confirm the presumed correlation between occurring hiperserotoninemia and constipation in those patients [123]. 

The gut microbiome can provide neurochemicals and neuropeptides for the host that can diffuse throughout the mucous layer of the intestine [128]. For instance, GABA can be released by *Lactobacillus* spp. and *Bifidobacterium* spp. [129]. Moreover, autistic children exhibit increased levels of gamma-amino-butyric acid (GABA), an inhibitory neurotransmitter [130]. Autistic brains are shown to demonstrate excitatory-inhibitory (E-I) imbalance. Disruptions in GABA activity in neurons are hypothesized to contribute to the pathogenesis of the disease [131].

### 6.3. HPA Axis 

The hypothalamus–pituitary–adrenal (HPA) axis is a critical component of the neuroendocrine system and plays a significant role in the body’s stress response [132]. The process initiates with the hypothalamus releasing corticotropin-releasing hormone (CRH) and stimulating the anterior pituitary gland to release adrenocorticotropic hormone (ACTH). In the bloodstream, ACTH reaches the adrenal cortex which stimulates the secretion of glucocorticoids, predominantly cortisol [133]. The HPA axis action on the caudal-intestinal axis is bidirectional [103]. While acute stress, mediated by cortisol might temporarily impact gut mobility and secretion, chronic stress may cause changes in the gut microbiota composition, modulate the immune response, and increase the permeability of the intestinal barrier [134]. Moreover, stress-related factors, including dysbiosis, can potentially activate and dysregulate the HPA axis function, thereby inducing stress response and anxiety-like behavior [135]. 

### 6.4. GALT

**Figure 3:**
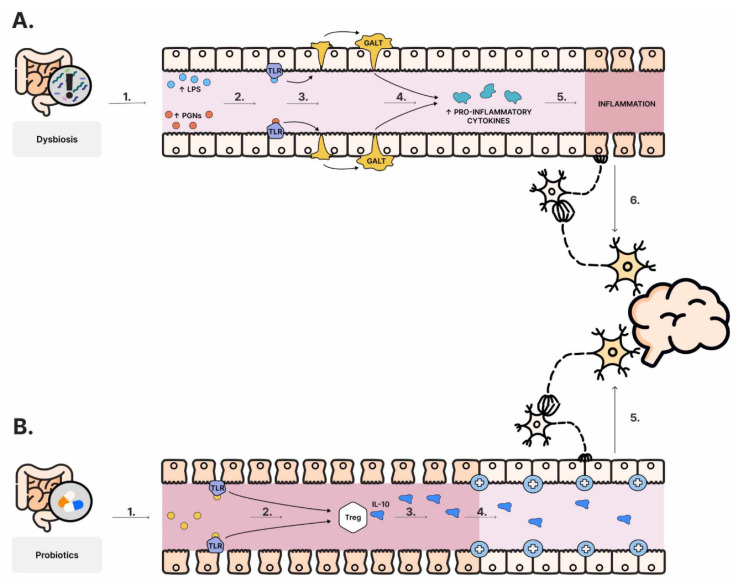
Diagram showing the link between gut bacteria and neuroinflammation. (**A**). Dysbiosis causes the release of lipopolysaccharide (LPS) and peptidoglycans (PGNs) (1). The molecules bind to Toll like receptors (TLRs) (2). Gut-associated lymphoid tissue (GALT) activation occurs (3). This leads to increased production of pro-inflammatory cytokines (4) and inflammation (5). Information is transmitted to the enteric nervous system (ENS) (6) and to the central nervous system (CNS). (**B**). Probiotics also secrete molecules that bind to TLRs (1), which affects the activation of regulatory T cells (Tregs) (2), secreting IL-10 (3), reducing inflammation (4) and transmitting the information to the ENS (5) and then the CNS.

The immune system plays a significant role in maintaining homeostasis between its defensive role, protecting the organism from externally derived pathogens, and simultaneously tolerating beneficial commensal organisms [136]. This is a crucial aspect as gut microbes actively contribute to the maturation of the immune response [23]. Additionally, emerging studies propose a link between gut bacteria, neurodevelopment, and neuroplasticity [137]. This connection is based on a delicate balance of pro-inflammatory and anti-inflammatory responses. Gut-associated lymphoid tissue (GALT) samples bacterial antigens and communicates with the ENS through signaling molecules, including cytokines and chemokines [138]. Bacterial products such as lipopolysaccharides (LPS) and peptidoglycans (PGNs) mediate the immune response through Toll-like receptors (TLRs) and deliver the information to the ENS [139]. The dysregulation of immune activation in the intestine, including those caused by dysbiosis, holds the potential for systemic inflammation, potentially influencing the CNS and consequently the mood, behavior, and cognitive function [85]. Furthermore, the anti-inflammatory effect of the probiotics might be expressed through the secretion of IL-10 by T regulatory cells [140]. Considering all this, the immune system’s multifaceted role in the gut–brain axis has implications for both physical and mental well-being (Figure 3).

## 7. Gastrointestinal Challenges in ASD Patients

### 7.1. Gastrointestinal Symptoms in ASD

Numerous studies highlight a higher prevalence (between 17% and 86%) of gastrointestinal (GI) symptoms in individuals diagnosed with autism spectrum disorder (ASD) compared to those with typical neurodevelopment [57,141]. The predominant symptoms occurring in ASD patients are diarrhea and constipation [141,142]. Additionally, individuals with ASD more frequently experience abdominal pain, vomiting, bloating, and gastric reflux [143]. However, presently the diagnostic and treatment approaches for gastrointestinal disorders are analogous to those in patients without ASD [144]. Nevertheless, certain studies do not point to differences in occurring GI symptoms between ASD patients and the control groups [145,146]. Some authors suggest that the lack of uniformity in findings might be a result of the existence of different phenotypes in the population of ASD patients [147].

It is worth noticing that some of the behavior problems, such as self-harm, aggression, sleep disturbances, and irritability might be a result of GI discomfort, especially since patients have trouble with verbal communication [113]. Speech disorders and intellectual impairment make it particularly troublesome to communicate abnormalities in GI physiology, which often results in numerous undiagnosed patients [148].

Alterations in gut bacteria composition may cause gastrointestinal discomfort, constipation, and diarrhea [48]. Dysbiosis may result from a selective and nutritionally inadequate diet with low fiber content. Children affected by ASD demonstrate various feeding disorders and are often described as “picky eaters”, representing food refusal and various dietary preferences [149,150]. Food selectivity manifests as texture, appearance, taste, smell, and temperature aversion [149,151]. Additionally, children with ASD manifest a strong preference for high-calorie snacks, and processed foods. Rejection of fruits and vegetables results in insufficient dietary fiber intake and often leads to constipation [152,153].

### 7.2. Malnutrition 

Improper diet results in health issues (e.g., malnutrition) and nutrient deficiencies, but also excessive body weight due to consumption of calorie-dense products [153]. What is more, inadequate intake of micronutrients (e.g., iron, calcium, vitamin E, and vitamin D) is common among these patients [154]. Some feeding disturbances are associated with poor oral intake [155]. Fastidious diet is one of the possible reasons for the previously mentioned lowered concentration of *Lactobacillus* spp., and consequently disturbances in forming tight junctions and maintaining the integrity of the gut barrier [55]. However, it is not fully discovered if nourishment difficulties cause dysbiosis and GI symptoms or if they are an additional factor influencing pre-existing dysbiosis.

### 7.3. Gastrointestinal Diseases

The higher incidence of gut dysbiosis and gastrointestinal symptoms in ASD patients raised a question about the increased prevalence of other GI diseases. Wang et al. in a study performed on children with autism indicated a higher prevalence of small intestinal bacterial overgrowth (SIBO) compared to children with typical neurodevelopment (31.0% compared to 9.3%). Additionally, the incidence of SIBO was associated with the severity of autism symptoms evaluated with the Autism Treatment Evaluation Checklist (ATEC) score [156]. This evidence needs further research; however, it suggests an influence of GI microflora on symptomatology and severity of ASD.

Due to common fundamentals in the pathogenesis of ASD and Inflammatory Bowel Disease (IBD) and the involvement of the microbiota–gut–brain axis, this subject became a research interest. In a retrospective case–cohort study conducted by Lee et al. on 48,762 autistic children and 243,810 controls in the United States, it was demonstrated that children with ASD have a greater probability of meeting the diagnostic criteria for subtypes of IBD, specifically Crohn’s disease (CD) and ulcerative colitis (UC), compared to healthy controls [157]. 

Researchers also considered the correlation between parental irritable bowel syndrome (IBS) and the occurrence of autism in their offspring. Sadik et al. in a nationwide population-based cohort study using Swedish registers represented evidence linking parental IBS with ASD diagnosis in children [158]. Moreover, this association was significantly stronger for maternal than paternal IBS diagnosis. Nonetheless, Andersen et al. in a registry-based nationwide cohort study including 1,005,330 children in Denmark did not find evidence of increased ASD prevalence among offspring of IBS-diagnosed parents [159]. Similar results emerged from the study performed by Yeh et al. in which researchers did not prove the association mentioned above [160]. Future research is required to strengthen the association between ASD and IBD and to better understand the role of the gut–brain axis.

### 7.4. Conclusions

Key information on gastroenterological problems in ASD patients is summarized in Table 1.

Considering the concept of “the microbiota–gut–brain axis”, dysbiosis can influence GI and neuropsychiatric symptoms. Some authors suggest a correlation between the disturbances in gut microbiota composition and the severity of autistic symptomatology, including verbal and social skills and general behavior [46,161]. Furthermore, unusual dietary patterns may favor harmful, pathogenic intestinal microbiota [162,163]. However, further research will be beneficial for a better understanding of existing interconnection.

The composition of gut microbiota is unique for each person and may change throughout life due to dietary and lifestyle changes. The foundation of a healthy microbiota lies in maintaining a harmonious balance and diversity. Further research should be performed to better understand potential advantageous approaches, including probiotic and prebiotic supplementation, along with fecal microbiota transplantation. Additionally, dietary intervention might be beneficial for maintaining homeostasis of the gut–brain axis, especially in ASD patients. 

## 8. Maternal Microbiome Dysregulation

In recent decades, attention has been drawn to maternal factors such as infections or injuries and their importance in influencing prenatal brain development [9]. Some epidemiological studies indicate an association between maternal infection and the child’s risk of schizophrenia and autism spectrum disorder (ASD) [164,165,166].

Some findings have shown a correlation between the occurrence of ASD in a child and prenatal infection with viruses: Herpes simplex virus type 2, Rubella, Cytomegalovirus, and bacterial infections [9]. However, the outcome of exposure to prenatal viral infection depends on many factors, including the maternal immune status, the strain and amount of virus, susceptibility of the maternal and fetal host, the developmental stage of the fetus, genetics, and probably other factors [167]. Some data did not find an overall association between maternal infection during pregnancy and diagnosis of ASD in the child. However, the same studies have observed an association between viral infection during the first trimester and bacterial infection during the second trimester and the development of ASD in the child [9]. 

Moreover, not specific pathogens but pathogen-induced maternal immune activation (MIA), has been considered as a key factor contributing to abnormalities in brain development and offspring’s behavior [168,169]. Inflammation during pregnancy possibly affects the proper proliferation and migration of neurons and glia, formation of synapses, myelination, and establishment of neuronal circuits [170,171]. What is more, environmental and general physical and social health factors like obesity [172], pollution [173], diet [174], poverty [175], and stress [176] can alter the immune system causing heightened inflammation, what can induce maternal immune activation and modify fetus neural development.

### 8.1. Maternal Immune Activation

Maternal immune activation (MIA) resulting from infection, stress, and autoimmune diseases is an increase in the levels of inflammatory markers during pregnancy [177]. Maternal inflammatory factors induce the release of pathogen-associated molecular patterns (PAMPs) and damage-associated molecular patterns (DAMPs), which activate Toll-like receptors on maternal immune cells and placental cells, leading to proinflammatory cytokine production [178,179]. Abnormal brain cytokine levels can alter CNS development [180]. Many studies using poly I:C and bacterial mimic lipopolysaccharide (LPS) to trigger MIA, have shown adult behavioral abnormalities in social behavior and selective attention, exploratory behavior, and working memory similar to ASD behaviors [181,182]. Cytokines could be produced directly in the fetal brain or cross an immature blood–brain barrier (BBB) [183]. Moreover, inflammation during neurodevelopment can cause damage to the blood–brain barrier and it could result in the loss of highly vulnerable neurons like dopaminergic cells [184] and may cause focal white matter injury [91].

### 8.2. MIA and Cytokines

Maternal immune activation is apparently disrupting the balance between pro-inflammatory and anti-inflammatory cytokines in the fetal brain [10,185]. It has been shown that pregnant women exposed to MIA may have dysregulated cytokine production, such as for interleukins: IL-6 and IL-17a, which is associated with offspring cognitive impairment [186,187]. 

### 8.3. IL-6

Elevated concentration of *C*-reactive protein during pregnancy is linked to the increased risk of ASD in the child [188]. This protein is synthesized by hepatocytes in response to IL-6 and other cytokines, such as interleukin-1β and TNF-α [189]. In the study by Smith et al., elevated levels of IL-6 were found in MIA models in the maternal serum, as well as in the amniotic fluid, placenta, and fetal brain. They found that IL-6 is a key mediator of the effects of maternal immune activation on fetal brain development mediating the behavioral and transcriptional changes in the offspring [186]. MIA affects gene expression of spanning gene families affecting cell structure and function such as cytosolic chaperone system, HSC70, Bicaudal D, aquaporin 4, carbonic anhydrase 3, glycine receptor, norepinephrine transporter, and myelin basic protein in the brains of the offspring [190]. MIA could also be induced by a single injection of IL-6, yielding offspring with the same behavioral abnormalities seen in viral infection [186]. 

**Figure 4:**
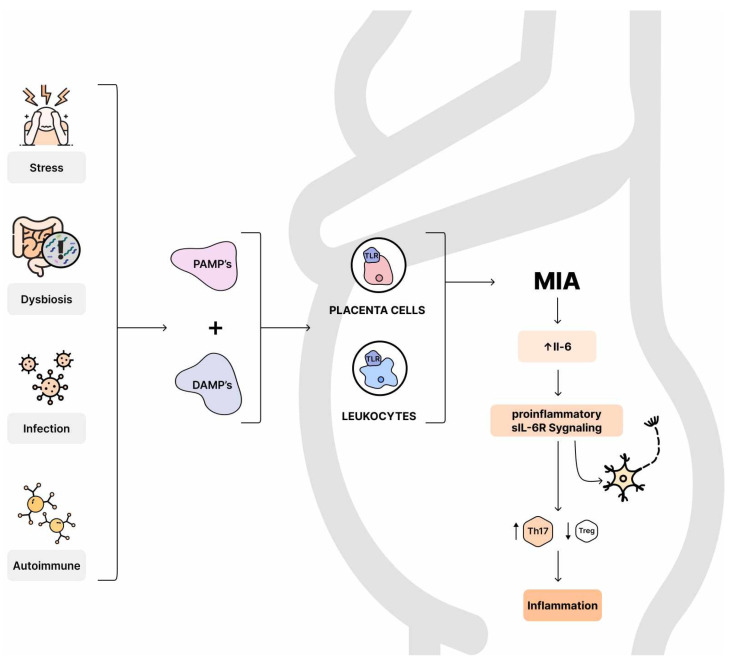
Stress, autoimmune diseases, infections and dysbiosis promote the release of pathogen-associated molecular patterns (PAMPs) and damage-associated molecular patterns (DAMPS), binding to TLR receptors on maternal leukocytes and placental cells, resulting in maternal immune activation (MIA). Increased IL-6 concentration affects neural cells via pro-inflammatory sIL-6R signaling, simultaneously promoting an inflammatory response with a predominance of Th17 lymphocytes over immunosuppressive regulatory T cells.

However, it is worth noting that IL-6 is considered both anti- and to a lesser extent proinflammatory cytokine [191]. The regenerative or anti-inflammatory function of IL-6 is mediated by classic signaling, whereby only a few cells express the IL-6 receptor and respond to it. Pro-inflammatory responses of interleukin-6 are rather mediated by trans-signaling (sIL-6R) [191]. Neural cells and neural stem cells depend on the sIL-6R in their response to IL-6 [192,193], which suppresses Treg cells and induces the differentiation of effector TH17 cells [194]. Differentiated from naive cells under the influence of IL-6, Th17 cells are involved in autoimmune processes and inflammation, while Treg inhibits excessive effector T cell responses [195] (Figure 4).

Moreover, Hei et al. showed that blocking the trans-signaling of IL-6 increased sociability in mice and induced glutamate release in synaptoneurosomes from the cerebral cortex [196]. Additionally, some data indicate that the adoptive transfer of regulatory T cells reverses behavioral phenotypes associated with autism. It may upregulate anti-inflammatory IL-10 and enhance chemotaxis and brain infiltration [197]. As well co-administration of an anti-IL-6 antibody in pregnant exposed to MIA triggers prevented behavioral deficits in offspring [186].

### 8.4. IL-17a 

It is considered that elevated IL-17a levels may be related to the severity of behavioral symptoms in individuals with ASD [198]. As described above, MIA increases maternal IL-6 levels, while the Th17 cells activated by IL-6 increase IL-17a production [199,200]. It has been detected that IL-17a mRNA levels are elevated in the placenta in response to MIA [11]. 

IL-17a aids in the tissue repair process, induces innate immune-like defenses by promoting the production of antimicrobial peptides and it has also been associated with its pro-inflammatory role in autoimmune diseases [201]. This cytokine performs also its function in the brain: it controls synaptic plasticity and short-term memory by increasing the glutamatergic synaptic plasticity of hippocampal neurons [202] and elevated concentration may elicit brain endothelial damage and cognitive dysfunction [203]. 

MIA promotes the activation of maternal Th17 cells without concomitant activation of Tregs [11], which unbalances the placenta and may promote fetal abnormalities [204]. 

### 8.5. Cytokine Imbalance in ASD

Cytokine imbalance and increased proinflammatory interleukins are also characteristic of children diagnosed with ASD [198,205]. Aberrant expression of cytokines was observed in the brain [91], peripheral blood [206,207] and gastrointestinal tract [208]. Among the various cytokines characteristic was an increased level of IL-1B, IL-6, IL-4, IFN-γ, and TGF-B [206,209,210,211,212]. 

However, cytokines expression is dependent on genetic and environmental factors [205]. Interleukins: -1, -2, and -6 have been shown to modify neuronal release of norepinephrine (NE), serotonin (5-HT), dopamine (DA), and acetylcholine (ACh) in the hippocampus and other brain regions such as striatum and frontal cortex [213,214].

The study by Hornig et al. has shown neuronal apoptosis in the hippocampus and cerebellum after inoculating Lewis rats intracerebrally with the Borna disease virus. Increased mRNA transcripts for IL-1α, IL-1β, IL-6, and TNF-α were observed in multiple brain regions in infected rats with behaviorally dysregulated exploratory activity [215].

Brain samples from patients with ASD revealed increased microglial activity with, at the same time, elevated levels of TNF-α, IL-1β, IL-6, IL-13, and C-C motif chemokine 2 (MCP-1) in the cerebrospinal fluid [91]. Elevated levels of TNF-α have been positively associated with the severity of ASD symptoms and they play a role in altering synaptic plasticity and glutamate-mediated cytotoxicity [216,217], which can cause apoptosis of hippocampal neurons [218]. 

The effects of maternal inflammation may induce long-lasting epigenetic memory on fetal microglia and immune cells during critical developmental periods [219]. In various models of MIA, hypomyelination, and degeneration of oligodendrocyte progenitor cells have been described [220,221]. 

The formation of mature neural circuits is caused by the selective elimination of inappropriate synaptic connections. Some immune molecules such as complement protein C1q are involved in pruning and refinement of the developing nervous system [222]. Among the cytokines, IL-6 has a stimulatory effect on C1q production in macrophages [223]. 

MIA can also disrupt neuronal migration during brain development [224]. Some MIA models have shown reduced expression of reelin, a major regulator of radial migration of cortical principal neurons [225]. Simultaneous low expression of reelin has been found in blood samples from patients with ASD [226].

### 8.6. Vitamin D in ASD

Vitamin D plays an important role in the process of early stages of brain development, neuronal differentiation, neurotransmission, and synaptic function [227]. Moreover, emphasis is placed on its role as a neuromodulatory and neuroprotective agent [228]. Decreased levels of vitamin D may influence the T cell activation profile and therefore adaptive immunity [229]. Stimulation of the tryptophan hydroxylase type 2 enzyme by vitamin D results in increased cerebral synthesis of serotonin [230].

A pivotal role in vitamin D involvement in the pathogenesis of ASD is played by vitamin D binding protein (DBP). DBP is encoded by the GC gene and involves different polymorphisms, which results in the binding of different vitamin D metabolites in the blood, regulation of serum concentration, and distribution in the body. Bolognesi et al. showed that the occurrence of GC1f genotype was more probable in ASD-diagnosed individuals, as well as with worse clinical symptoms [231]. While acknowledging the limitations of this study, it is important to emphasize the need for more extensive investigations involving larger groups of both patients and controls, since this research area holds promise for the discovery of genetic markers associated with specific ASD phenotypes.

Chronic activation of microglia might cause damage to the central nervous system and is commonly mentioned in the context of ASD pathogenesis. It is noteworthy that microglia express vitamin D receptors. Boontanrart et al. demonstrated in mouse models that vitamin D binds to the vitamin D receptor (VDR) and by that reduces the release of pro-inflammatory cytokines (IL-6, IL-12, and TNF-α), and increases the expression of anti-inflammatory IL-10 [232]. 

Vuillermot et al. in the mouse models of inducted MIA (maternal immune activation) prenatally administered an active form of vitamin D which resulted in abolished ASD-like behaviors (e.g., stereotyped digging and social withdrawal in the offspring). Nevertheless, vitamin D did not alter levels of pro-inflammatory cytokines [233]. Other study conducted by Guerini et al. indicated that decreased concentrations of vitamin D and VDR polymorphisms correlate with structural and functional brain abnormalities and behavior disturbances. Studies have also revealed that children with ASD and their mothers are more prone to vitamin D/VDR complex with low biological activity [234]. However, further research is required for a better understanding of vitamin D immunomodulatory properties and its importance in autism.

### 8.7. Gender Differences 

The importance of developing autism in offspring as a result of excessive activation of the mother’s immune system is enhanced by increased concentration of antibodies compared to mothers of healthy children [235].

Bauman et al. isolated IgG from mothers of children with ASD (IgG-ASD) and administered it to two groups of female rhesus monkeys during the first and second trimesters of pregnancy. Developmental abnormalities were observed in monkeys exposed prenatally to human IgG-ASD. Male offspring had enlarged brain volume compared with controls and these differences were most noticeable in the frontal lobes, while no differences in female subjects were identified [236]. However, it is important to indicate the limitations of the presented research. Among the limitations, it is noteworthy that the study sample size is small, and the animal model, although it can be a model for human neuropsychiatric disorders due to similarities in brain organization and observable behavioral changes similar to ASD, nevertheless limits the conclusiveness of conclusions. These data indicate that there are differences in the neuropathology of boys and girls with ASD. 

Other studies report a striking preponderance of males in terms of the prevalence of ASD [237]. However, there are gender differences in the manifestation of autism—males with ASD more often show externalizing behavior problems like aggressive behavior, hyperactivity, social behavior disturbances, and increased restricted interests. Females with ASD show more internalizing symptoms, including anxiety, depression, and other emotional symptoms [238]. Therefore, women may be under-diagnosed due to less clear symptoms. However, it is important to note that some studies suggest the meaning of female protective factors such as lower concentrations of testosterone, a hormone that plays a key role in the Extreme Male Brain theory—theory explaining brain masculinization as a cause of autism [239]. In brain regions which are on average larger in men than in women, i.e., amygdala or cerebellum, people with autism have larger brain areas than typical men, and their total brain volume is also larger than that of a neurotypical male control group [240]. 

What is more, even though ASD is not an X-linked disorder, sex chromosomes may modulate ASD risk as it is indicated by the higher prevalence of autism in: Klinefelter syndrome (XXY) [241], XYY syndrome but not in X chromosome trisomy (XXX) [242,243]. The study by Bishop et al. noted an association of autistic traits in males with an extra sex chromosome, with more noticeable changes observed in males with the XYY karyotype than XXY. Moreover, in this study, although girls with karyotype XXX also had a high rate of educational difficulties and required speech therapy, none of them had a diagnosis of ASD or significant communication problems [242]. 

However, the increased prevalence of autism in Turner syndrome [244] may seem contradictory to the above results.

Nevertheless, Creswell et al. conclude that the key is not the presence of the X-gene, but that there is an imprinted locus on the X-chromosome, which is inherited from a father and lowers the threshold for the phenotypic expression of genes that predispose to autism elsewhere on the genome. All patients with Turner syndrome and a diagnosis of autism in the above study either had no X-chromosome inherited from their father or it was abnormal. Boys, on the other hand, inherit the X chromosome only from the mother, so according to Creswell et al.’s hypothesis, a normal paternal chromosome may reduce a child’s risk of autism, so boys are potentially at higher risk for the disorder. However, it should be noted that genes effects are often pleiotropic and affects various other genes and their expression. The study’s final conclusion is that changes in genes on the X chromosome are not directly related to the traits of the autistic phenotype, but that its action affects other genes elsewhere in the genome that confer susceptibility to the development of the autistic phenotype [245]. In summary, imprinted locus on the X-chromosome still requires further study, and it has not been clearly determined why the threshold for autism expression in males is lower than in females. Furthermore, it should be noted that most children with autism spectrum disorders have a normal karyotype [246]. 

Nevertheless, the findings point to the possible importance of genes present on the sex chromosomes, with attention being paid to neuroligin (NLGN) genes, which encode proteins responsible for the formation of functional synapses, and the disruption of which has been linked to the occurrence of autism [247]. Moreover, deletion of the Xp22 fragment may be a potential cause of autism, since one of the neuroligin genes, NLGN4X, is present in this chromosome fragment [248]. Thus, a possible explanation for the increased risk of autism in children with sex chromosome abnormal syndromes is that genetic syndromes lacking the protective effects of the second X chromosome with properly functioning NLGN genes may have dysfunctional adhesion molecules involved in synapse formation and neurodevelopment. However, this study was performed on small groups, so it needs to be analyzed in more detail.

Tartaglione et al. also reported that a single injection of poly I:C to pregnant mice causes deficits in social interaction, changes in gut microbiota composition, and neuroinflammatory response in both sexes. However, males seemed more disturbed than females [249]. The effect of microbiota on microglia function in offspring is related to gender and age, with a greater impact in males during prenatal development, while in females during adulthood [250]. However, sex differences in microglia and microbiota still remain unknown [249]. 

In conclusion, maternal immune activation may increase pro-inflammatory cytokines, which may affect the microglia that shape normal neurogenesis in the first weeks of life. Numerous models point to the association of neuroinflammation with the potential development of autistic behavior, and therefore inhibiting the inflammatory response may be an important therapeutic target.

## 9. MIA and Microbiome Dysregulation

Animal studies indicated that in addition to genetic predisposition, the maternal gut microbiota may play an essential role in the occurrence of autism in offspring [110,251]. Maternal microbiome exposed to fat produces proinflammatory bacterial metabolites that can activate maternal innate immune cells, which may be the cause of MIA and the degenerative effect of this phenomenon on fetal neurodevelopment [251].

### 9.1. MIA and Impaired Intestinal Integrity

MIA offspring display behavioral features of ASD [252].

The study by Hsiao et al. showed that the offspring of mice with induced MIA, exhibit impaired intestinal integrity and changes in the composition of the intestinal microflora, reminiscent of those described in patients with ASD. Deficits in intestinal integrity were detectable in 3-week-old MIA offspring, and in adult MIA offspring in both the small and large intestine, insufficient expression of the ZO-1 (Zonula occludens-1) gene, which encodes scaffolding protein that crosslinks and anchors Tight Junction (TJ) strand proteins, was detected. What is more, the same study has shown that *Bacteroides fragilis* supplementation corrects intestinal permeability in MIA offspring and alters the expression of genes that regulate intestinal barrier integrity [110]. However, an excess of certain strains of *B. fragilis*, on the contrary, can reduce integrity due to the secreted fragilinase, a metalloproteinase capable of cleaving E-cadherin, which binds epithelial cells together [253]. 

Another study also in an animal model confirms increased intestinal integrity with supplementation of *B. fragilis* strains, while showing that they can promote stem cell regeneration and increase mucus secretion in intestines [254]. The positive effect on sealing the intestinal barrier can be linked to the effect of these bacteria on increased synthesis of SCFA, anti-inflammatory IL-22 and promoting the development of regulatory T cells [255]. 

Simultaneous oral treatment with *Bacteroides fragilis* and *Bacteroides thetaiotaomicron* may improve communicative, repetitive, anxiety-like, and sensorimotor behavior associated with ASD in mice [110]. However, more recent studies do not support this link, and in fact indicate that dysbiosis in autistic individuals is characterized by elevated levels of *Bacteroides* spp. which in animal models can worsen behavior [256]. The increase in this strain is confirmed by meta-analyses using several cohorts, with concomitant elevated IL-6 levels in ASD patients [257]. 

What is more, Hsiao et al. found that *B. fragilis* lowers IL-6 levels in the colon [110]—an interleukin with an elevated level characteristic of MIA and may be related to behavioral deficits in autism [186]. Interestingly, IL-6 and other cytokines regulate tight junction expression; and, at the same time, the microbiome regulates cytokine levels [258]. Recent studies also point to the anti-inflammatory effects of *B. fragilis* in the gut. Due to *B. fragilis* administration, levels of colonic pro-inflammatory cytokines such as TNF-α, IL-1β, and IL-6 were reduced and IL-10 increased [259]. 

### 9.2. Gut Microflora and MIA

Maternal immune activation may be directly related to the gut microbiome. Kim et al. found that supplying vancomycin to the offspring of mothers injected with poly I:C, prevented them from induction of behavioral abnormalities in MIA offspring. Moreover, it led to lower levels of maternal IL-17a and offspring did not develop cortical lesions [187]. This may indicate that vancomycin-sensitive gut microflora play a role in stimulating cells to IL-17a production and for the development of MIA-related behavioral abnormalities in the offspring [260]. It is worth mentioning that vancomycin’s spectrum of action includes bacteria including *Clostridioides* spp., the concentration of which may be elevated in ASD and is one of the hypotheses of its pathogenesis [261].

The adult gut microflora is individual and it has been considered to be stable throughout life [262]. However, more recent studies indicate that a change in diet can drastically affect the composition of the already-formed microbiota [263]. This leads to the question of whether the previously normal microflora of the mother under the influence of environmental factors such as a diet rich in fats, can change and can be passed on to the child, which, with the presence of appropriate genetic factors, can develop ASD. 

Dysbiosis can cause activation of inflammation, leading to a mechanism resembling a viral infection and resulting in maternal immune activation, which can result in behavioral changes in the offspring. This may be related to both impaired intestinal junctions and metabolites and molecules produced by the bacteria, which can stimulate the mother’s immune system even without an ongoing infection. Unambiguous identification of gut microflora disorders in ASD is difficult for several reasons. One is, on the one hand, the discernible differences in the composition of the intestinal microflora of children with ASD relative to a sex- and age-matched neurotypical control group while, at the same time, the not so clearly discernible difference between neurotypical siblings and children with ASD. Here, the differences may be due to a different age group and gender [257]. 

At the same time, some studies indicate that siblings of children with ASD are more likely to develop autism [264]. Further research is also needed to analyze why gender variance with male dominance is characteristic in behavioral changes in animal models. 

## 10. Obesity and a High-Fat Diet 

### 10.1. Mother’s Obesity and ASD

Among the pregnancy factors that increase the risk of ASD is maternal obesity [265]. Obesity, insulin resistance, and type 2 diabetes are associated with systemic and adipose tissue inflammation [266]. Gut microbiota may alter adipose tissue inflammation and impair glucose metabolism due to being a source of pro-inflammatory molecules such as lipopolysaccharides and peptidoglycan [267]. Plasma lipopolysaccharide levels increase with a higher fat diet in mice [268] and humans [269]. A high-fat diet may increase the percentage of gut microflora containing LPS, which leads to an increase in inflammatory markers and triglycerides in the liver [268]. 

Moreover, both obese mice and humans have an altered composition of intestinal microflora—microbiota contains more *Firmicutes* and fewer *Bacteroidetes* [270]. *Bacteroidetes* may respond to caloric intake due to an increase in their amount under the influence of a change to a low-fat, low-carbohydrate diet [271]. The abundance of bacterial strains is also influenced by the supply of prebiotics—inulin increases the levels of *F. prausnitzii* and *Bifidobacterium* spp. in humans [272].

### 10.2. A Maternal High-Fat Diet (MHFD)

A maternal high-fat diet (MHFD) may induce behavioral modifications in offspring due to chronic low-grade inflammation [273], macrophage recruitment, and increased pro-inflammatory cytokines in adipose tissue [274] or changes in maternal gut microbial ecology [275]. Hildebrandt et al. found that both obese and non-obese mice fed on high-fat diets have reduced numbers of *Bacteroidetes*, and increased numbers of *Firmicutes* and *Proteobacteria* [276]. Among several species especially abundance of *Lactobacillus reuteri* was diminished in MHFD offspring [251]. *L. reuteri* has been shown to promote oxytocin levels [277], a hormone that dysregulation is considered a potential cause of autism [278]. Oxytocin modulates behavior, learning, and memory [279]. In healthy individuals, administering oxytocin increases emotion-identification of human faces and attention to the eye region of faces [280]. Modahl et al. found that oxytocin levels do not increase before the onset of puberty in individuals with ASD, unlike neurotypical individuals [281]. Moreover, polymorphisms in the oxytocin receptor genes are related to symptom severity of ASD [282]. 

The study by Buffington et al. showed that MHFD offspring have fewer oxytocin immunoreactive neurons in the hypothalamus. What is more, they found that changes induced by diet in the offspring gut microbiota block long-lasting neural adaptation in the ventral tegmental area, which is a mesolimbic dopamine reward system. The data showed that probiotics may correct oxytocin levels and synaptic dysfunction in the VTA and reverse behavioral deficits in MHFD offspring [251]. 

What indicates the importance of the intestinal microflora is the fact that transferring non-MHFD gut microbiota between mice by the fecal-oral route corrected social deficits in MHFD offspring. However, there is a neurodevelopmental window during which microbial transfer improves behavior [251]. 

Maternal factors during pregnancy therefore have a significant impact on the formation of the developing fetus. Special attention should be focused on protecting the mother from contact with infectious agents, but this cannot be fully avoided. What the mother can influence, however, is to make sure she has a proper low-fat diet rich in fiber and vitamins to strengthen the role of commensal bacterial flora. Additionally, body weight reduction is advised to avoid chronic inflammation resulting from inflammation-active adipose tissue.

## 11. Therapeutic Targets

Considering gut–brain axis pathomechanism and abnormal gut microbiota in ASD patients, researchers took action to examine a variety of treatments. Their focus embraces probiotics, antibiotics, microbiota transfer therapy, digestive enzymes, and even helminth therapy. The aim is to modify the gut microbiota and therefore improve GI and behavioral symptoms among children with ASD.

### 11.1. Probiotisc and Prebiotics 

Many studies have been performed to show the efficiency of probiotics in ASD patients, as these have therapeutic effects on animals. It was proven that probiotics mixture, which is *Lactobacillus* spp. and *Bifidobacterium* spp. improved social ability in (the validated valproic acid- or antibiotics-induced) animal model [283]. 

The efficiency of probiotics in ASD human patients was confirmed by Lauren M. Schmitt et al.: They reported that a formulation of *L. reuteri*, dextran microparticles, and maltose improves adaptive behavior and social preference and remains well tolerated [284]. 

In another study, through probiotics and fructo-oligosaccharide intervention, the level of *Bifidobacteriales* and *Bifidobacterium longum* increased, while *Clostridioides* decreased. These alterations in gut microbiota were observed to elevate SCFAs, which can modulate the production of neurotransmitters serotonin and dopamine via the gut–brain axis, leading to improved ASD symptoms [285].

However, randomized clinical trials testing the effectiveness of probiotic and prebiotic therapy in children with ASD are still lacking. Overall, the evidence supporting the effectiveness of probiotics in alleviating gastrointestinal symptoms or behavioral disorders found in children with autism is limited [286]. 

What is more, findings of the systematic review and meta-analysis revealed no efficacy of probiotics supplementation in children with ASD. More high-quality studies on children are needed to examine the therapeutic effects of probiotics. Although animal studies seem to give great evidence of improving ASD-like symptoms, there are significant differences between mice and humans, that must be taken into consideration such as species dissimilarity, the drug’s role in inducing ASD model in mice, and complex pathomechanism of ASD in humans [287].

### 11.2. Antibiotics

When it comes to antibiotics, numerous studies indicate, that pre- and postnatal antibiotic exposure can be a risk factor for ASD in children, especially prenatal ones, which is associated with dysbiosis described above [288].

A study by Logan K. Wink et al. suggests, that D-Cycloserince (DCS) appears to improve social skills in ASD children compared to placebo but further studies are needed to see the long-term impact of DCS [289]. 

Minocycline is a broad-spectrum tetracycline antibiotic, which, primarily, is used to treat bacterial infections but also has a neuroprotective, anti-inflammatory, and antioxidant effect [290,291]. Hence, it became an object of interest as an adjunctive treatment for psychiatric and neurobiological conditions [292]. 

In one study, ASD children received minocycline for 10 weeks in addition to risperidone, as there is a lack of positive findings in efficacy of minocycline alone. As a result, there were improvements in subscales of irritability and hyperactivity/noncompliance. Due to the immunomodulatory and anti-inflammatory characteristics of minocycline, it is hypothesized to be an adjunctive treatment to risperidone in children with ASD [293].

### 11.3. Microbiota Transfer Therapy (MTT)

Microbiota Transfer Therapy (MTT) is a modified fecal microbiota transplant, which includes a bowel cleanse, two-week vancomycin treatment, commensal microbes from a healthy donor transplant, and stomach-acid suppressant. According to an MTT intervention on 18 ASD-diagnosed children The Gastrointestinal Symptom Rating Scale showed an approximately 80% improvement in GI symptoms and these remain for eight weeks following treatment. Furthermore, there was a major reduction in behavioral symptoms of ASD, and the gut microbiome was affected, in particular, increased *Bifidobacteria*, *Prevotella*, and *Desulvovibrio* [294]. Two years after the MTT trial, the same participants were re-evaluated and researchers still observed higher gut microbiota diversity, increased *Bifidobacteria* and *Prevotella*, and improvement in behavioral symptoms [295]. After comprehensive metabolomic measurements on plasma and fecal samples taken from previous clinical trial, it was found that, in the beginning, ASD children had different metabolite profiles in comparison to typically developing children.

Differences included lower levels of nicotinamide riboside, IMP, iminodiacetate, methyl succinate, galactonate, valylglycine, sarcosine, and leucylglycine and a higher level of caprylate and heptanoate. From being different at baseline, plasma metabolite profiles appeared to resemble those in the typically developing group. This shows, that MTT had a huge impact on plasma metabolites, and this way it can be a promising form of therapy for ASD children [296].

### 11.4. Enzymes

The study by Horvarth et al. shows a lack of digestive enzymes in autistic children. They observed low activity of at least one disaccharidase or glucoamylase, but in particular low levels of lactase and maltase, and this resulted in gaseousness and loose stools [297]. More studies have been taken to establish if introducing digestive enzymes can improve ASD symptoms, and the results are mixed. According to a study on 101 children with ASD, 3 months of digestive enzyme therapy can ease behavior and GI symptoms. There were improvements in socialization, hyperactivity, stool quality, stomach aches, vomiting, and food variety [298].

In a 12-month treatment study of a comprehensive nutritional and dietary intervention, which included special vitamin/mineral supplements, fatty acids, Epsom salt baths, carnitine, healthy gluten-free, casein-free, soy-free diet, and digestive enzymes the most effective were vitamin/mineral supplements, essential fatty acids, and soy-free diet as they have a role in improving nutritional status and nonverbal intellectual ability. As it comes to digestive enzymes they appear to have insignificant clinical benefits [299].

### 11.5. Helminth Therapy

Due to the fact that inflammatory conditions have been established in ASD as crucial factors, helminth therapy has become a subject of interest. Helminth supports anti-inflammatory Th2 immune response, reverting pro-inflammatory Th1 activation, sustaining gastrointestinal homeostasis, and also increasing production of IL-3, IL-4, IL-5, and IL-10 which can lower IL-6, IL-1B, and IL-12, increased in autism [300]. To examine the potential therapeutic effects of helminth a pilot study on ASD adults was conducted and the results show improvements in repetitive and restricted behavior. Although further studies are needed to confirm the beneficial side of this treatment, this study indicates that immune-modulating helminths may be a helpful therapy strategy for ASD patients in the future [301].

### 11.6. Maternal Therapeutic Targets

An attempt to influence the gut microbiota of the mother may be useful in potentially preventing the appearance of autism, reducing its symptoms, or facilitating diagnosis. Several studies indicate the possibility of using specific strains of bacteria as biomarkers in children with ASD and their mothers [7,302]. Possibly therapeutic targeting of Th17 cells in susceptible pregnant women may reduce the risk of bearing children with inflammation-induced ASD [11].

Attempts to use probiotics in pregnancy as a factor regulating disregulations in the composition of the mother’s microflora and the consequences of this condition on fetal neurodevelopment may also be promising [303].

The animal study by Xiao Wang et al. has shown that oral probiotic administration (*Bifidobacteria*, *Lactobacillus helveticus*, fructooligosaccharides, and maltodextrin) in female mice prevented MIA-Induced ASD-relevant deficits in adult offspring. What is more, it prevented parvalbumin-positive neuron loss, increased proinflammatory cytokines levels in maternal serum and fetal brain, and decreased GABA levels [304]. 

More careful use of antibiotics in pregnancy as a potential risk factor for autism in offspring may also be important [305]. Antibiotics release CW (bacterial cell wall peptidoglycan), which is a PAMP (pathogen-associated molecular pattern) for Toll-like receptors, especially TLR2. CW can transfer via the placenta to the fetal circulation and induce FoxG1 (neuronal transcription factor), which causes neuroproliferation in the cortex, leading to increased dell density and then, postnatal behavioral abnormalities [306]. Overexpression of FoxG1 induces GABAergic neuron overproduction and can be associated with an increased risk of autism spectrum disorder [307].

A study was performed to examine if exposure to broad-spectrum ampicillin antibiotic during a narrow critical perinatal window can induce ASD-like behaviors in mice. Researchers demonstrated reduced gene expression of the oxytocin receptor and tight-junction proteins in the prefrontal cortex, (which is responsible for social and emotional behaviors) in exposed juvenile males and it resulted in atypical behavioral symptoms [305]. However, the population-based cohort study consisting of 96,736 children aged 8 to 14 years showed a small increased risk for ASD after using various antibiotics during pregnancy [308]. Moreover, a study by Yu-Chun Lin et al. examines if there is a potential association between prenatal antibiotic exposure at a specific time and developing ASD in offspring. It was proven, that only exposure after 34 weeks of gestation increased the risk of ASD, but still slightly [309]. 

A small increase in risk was also observed in the study by Amanda S Nitschke et al., but particularly during the first and second trimesters [310]. In different cohort studies, there was a 10% increase in risk in those exposed to antibiotics, but in the second or third trimester [311]. 

Summing up, there are some conflicting results about the trimester, in which the risk of developing ASD after antibiotic exposure is the highest. Although antibiotics have been shown to have not such a significant role in causing ASD, more careful use during pregnancy can be suitable. Total withdrawing antibiotics during a bacterial infection can have much worse consequences for both mother and fetus [312]. 

### 11.7. Vitamin D Supplementation

Due to the immunomodulatory and neuroprotective role of vitamin D, its deficiency during pregnancy contributes to immune and behavioral anomalies in post- gestational age [313]. Vitamin D deficiency can cause pre-eclampsia, preterm birth and gestational diabetes and these increase the risk for ASD in newborn. Hence, it is hypothesized that vitamin D supplementation to pregnant women lower the risk of autism in the infants [314]. 

A comprehensive meta-analysis carried out by Wang et al. indicated the higher prevalence of lower concentrations of vitamin D in individuals with ASD but also establish a correlation between reduced vitamin D levels and an elevated risk of developing ASD. In addition, the analysis emphasized the impact of maternal and neonatal vitamin D concentrations revealing a pattern of decreased early-life levels in the ASD group. It indicated a potential link between diminished maternal or neonatal vitamin D and the predisposition to ASD development [315]. Moreover, Feng et al. observed that autistic children with lower vitamin D levels showed poorer language and behavioral performance, and after following 3 months of vitamin D supplementation improved their behavior, particularly among children under 3 years of age. Nevertheless, the study was conducted on a relatively small group. However, results suggest promising areas for further investigation [316]. 

A study by G. Stubbs et al. was conducted to show if supplementation with vitamin in pregnancy is beneficial in decreasing the risk of autism in the offspring and results were promising. Pregnant women with a previous ASD child received Vitamin D3 5000 IU/d, which is higher doses than is commonly recommended, followed by the supplementation of a newborn with Vitamin D3 1000 IU/d to their third birthday and only 5% children developed autism [317]. Furthermore, the study by Pérez-López et al. proved, that lower maternal serum levels of 25(OH)D appears during the first trimester and have been associated with ASD in offspring. Although it seems reasonable to start supplementation during this specific time, further studies are needed to confirm that statement [318].

Moreover, attention should be paid to the potentially dangerous effects of overdosing on vitamin D at higher than recommended doses. It is therefore necessary to monitor vitamin D blood levels to prevent toxicity and dangerous complications like hypercalcemia [319]. 

## 12. Limitations

The study is not a systematic review and does not provide quantitative information. Strict inclusion and exclusion criteria were not applied. Both large and small studies were included.

## 13. Conclusions

Changes in the gut microbiome of ASD patients have been confirmed in studies. The greatest importance of the mode of delivery on microbial diversity in the early period of a child’s life continues to be indicated, with exposure to the birth canal environment resulting in children born by vaginal delivery being populated with more diverse communities of *Lactobacillus* and *Bifidobacterium* as opposed to those born by cesarean section. These differences diminish and are less noticeable during the first weeks after birth, and it is worth noting that this time is crucial for the child’s neurodevelopment. The most important potential significance of the probiotic strains is summarized in Table 2.

Dysbiosis can increase inflammation in the mother’s and children’s bodies, with a high-fat diet, maternal obesity, or antibiotic therapy likely to disrupt the composition of the microflora. However, further well-designed studies are needed due to the limitation and inconclusiveness of the studies so far, resulting from the inability to verify the authenticity of the cases analyzed and conclusively determine whether the source of the disruption is genetic or an external factor. An additional limitation is the number of studies on animal models and cohorts relative to studies providing strong evidence. 

Nevertheless, it is not clear what is the main cause of intestinal dysbiosis in ASD patients—it is not fully discovered whether food selectivity, a characteristic symptom of the disorder, causes dysbiosis and gastrointestinal symptoms, or whether feeding difficulties are a factor in pre-existing dysbiosis.

Given the potential association of abnormal gut microflora in ASD patients, numerous studies have been conducted to evaluate various treatments, such as probiotics, antibiotics, microflora transfer therapy, digestive enzymes, and even helminth therapy to modify gut microflora and regulate inflammation. Probiotic therapies in children with ASD are promising, but more high-quality studies on children rather than animal models are needed to investigate the therapeutic effects of probiotics. Attempts to use probiotics in pregnancy as an agent to regulate maternal microflora composition and fetal neurodevelopment may also be promising. However, more data and broadcasts on human models are lacking, hampered by ethical motives and the difficulty of experimental studies on pregnant women. Likewise, the results of the effects of probiotic therapy should also be treated with caution due to the often inconclusive effects of bacterial strains on intestinal barrier tightness, the behavior of individuals in animal models and differences in the effects of probiotic therapy in clinical trials.

As for antibiotics, several studies suggest that exposure to antibiotics before and after birth may be a risk factor for ASD in children, but the data are conflicting. Extremely promising is microbiota transfer therapy (MTT), which affects changes in plasma metabolites and may be an effective therapy to modify autistic symptoms. Also new is research into helminth therapy, which supports an anti-inflammatory immune response and reduces inflammation. Several studies also point to the possibility of using specific bacterial strains as biomarkers in children with ASD and their mothers. 

Overall, special attention should be paid to environmental factors modifying the composition of the mother’s microflora and the course of her inflammatory response. Further research is needed to address issues such as the composition, optimal dose, and duration of probiotic supplementation in children with ASD, but also well-designed studies evaluating probiotic therapy as well as antibiotic therapy in pregnancy. Specific bacterial strains or their metabolites could be helpful in the early diagnosis of ASD as an early marker of autism. Careful analysis of gastrointestinal symptoms in children with ASD could also speed up diagnosis and early introduction of therapy, especially in patients presenting with low-intensity symptoms. All of these measures would help develop effective treatment, prevention, and diagnostic strategies to improve ASD symptoms.

## Figures and Tables

**Table 1:** Autism spectrum disorder (ASD) and gastrointestinal problems.

Frequency	Symptoms	Gastrointestinal Disorders
between 17% and 86% of gastrointestinal symptoms in individuals diagnosed with ASD [57,141]	Predominant: diarrhea and constipation [141,142] Other: abdominal pain, vomiting, bloating, and gastric reflux [143] feeding disorders—often described as “picky eaters” [149,156]	Higher prevalence of small intestinal bacterial overgrowth (SIBO) Crohn’s disease (CD) and ulcerative colitis (UC), compared to healthy controls [157]

**Table 2:** The potential role of the bacteria.

*Bifidocaterium*	*Lactobacillus* spp.	*Bacteroides* spp.
producing SCFAs [92,93,94] GABA releasing [129] preventing MIA-Induced ASD-relevant deficits in adult offspring [304] preventing parvalbumin-positive neuron loss, increased proinflammatory cytokines levels in maternal serum and fetal brain, and decreased GABA levels [304]		
intestinal refilling of tryptophan (*Bifidobacterium infantis*) [120]	inducing changes in γ-aminobutyric acid (*L. rhamnosus*) [107] taking part in maintaining tight junctions between cells [55] preventing the overgrowth of Candida [56] promoting oxytocin levels (*L. reuteri*) [277]	*B. fragilis: * regulating intestinal barrier integrity [110] lowering Il-6 in colon [110,259] reducing gut permeability [53] promoting stem cell regeneration and increase mucus secretion in intestines [254] promoting the development of regulatory T cells and IL-22 secretion [255] ⬆ *B. fragilis* may reduce integrity due to the secreting fragilinase [253] *Bacteroides* spp.: improving communicative, repetitive, anxiety-like, and sensorimotor behavior associated with ASD in mice (*Bacteroides thetaiotaomicron*) [110] ⬆ levels in autistic individuals [257] exacerbate social behaviors in mice [256]
